# Patient-Reported Outcomes Before and After Radiotherapy for Brain Metastases—A Prospective Cohort Study of 239 Non-Small-Cell Lung Cancer Patients

**DOI:** 10.3390/cancers17091529

**Published:** 2025-04-30

**Authors:** Astrid Telhaug Karlsson, Marianne Jensen Hjermstad, Nina Aass, Eva Skovlund, Guro Lindviksmoen Astrup, Stein Kaasa, Olav Erich Yri

**Affiliations:** 1 Regional Advisory Unit for Palliative Care, Department of Oncology, Oslo University Hospital (OUH), 0450 Oslo, Norway; mariajhj@medisin.uio.no (M.J.H.); naa@ous-hf.no (N.A.); gurol@ous-hf.no (G.L.A.); stein.kaasa@medisin.uio.no (S.K.); olavy@ous-hf.no (O.E.Y.); 2 European Palliative Research Centre (PRC), Oslo University Hospital (OUH), 0450 Oslo, Norway; 3 Department of Oncology, Oslo University Hospital (OUH), 0450 Oslo, Norway; 4 Institute of Clinical Medicine, University of Oslo, 0025 Oslo, Norway; 5 Department of Public Health and Nursing, Faculty of Medicine and Health Services, NTNU—Norwegian University of Science and Technology, 7491 Trondheim, Norway; eva.skovlund@ntnu.no

**Keywords:** non-small-cell lung cancer, brain metastases, radiotherapy, patient-reported outcomes, quality of life, palliative care

## Abstract

**Introduction:** Radiotherapy (RT) is a frequently offered treatment option for brain metastases (BMs) in patients with non-small-cell lung cancer (NSCLC). This study presents patient-reported outcomes (PROs) in a cohort of NSCLC with BMs treated with RT. This study researched how PRO scores at the start of RT may be useful in survival estimates and how PROs change over time after RT. **Methods:** NSCLC patients with first-time BMs treated with RT were identified in a prospective observational study. PROs were collected at the start of RT and monthly for up to 1 year. Differences in PRO mean scores at the start of RT (M0) and at month 2 (M2) after treatment are reported. Prognostic values of PROs were analyzed in a stepwise adjusted Cox model. **Results:** Of 294 patients identified, 239 (81%) responded at M0; 105/239 (44%) responded at both M0 and M2. High scores for weakness of legs at M0 were associated with short survival when adjusting for performance status and status of extracranial metastases. Those responding at M0 only had worse mean scores for overall QoL and PF but similar scores for fatigue and dyspnea compared to patients responding over time. At M2, patients with <6 months survival after RT reported worse scores for overall QoL, PF, fatigue, and dyspnea; long-term survivors reported stable scores. **Conclusions:** NSCLC patients diagnosed with BMs and expected survival < 6 months should be offered optimal palliative care rather than RT.

## Simple Summary

Brain metastases occur in up to 40% of patients with non-small-cell lung cancer (NSCLC). The majority of patients are treated with radiotherapy. Patient-reported outcomes (PROs) provide information directly from the patients about their perceived health status, level of function, and symptom burden. The aim of this study was to identify patients that are unlikely to benefit from radiotherapy. We report PRO scores at the start of radiotherapy and changes in scores after two months in a prospective study cohort of 294 NSCLC patients. Patients with survival <6 months after radiotherapy frequently reported worse PRO scores two months after treatment whereas those who survived >6 months generally reported stable scores. Based on PRO scores, NSCLC patients with brain metastases and a short expected survival do not seem to benefit from radiotherapy. They should rather be offered optimized palliative care as a better treatment alternative.

## 1. Introduction

During the course of the disease, up to 40% of patients with non-small-cell lung cancer (NSCLC) develop brain metastases (BMs). This rising incidence is due to improved survival with new systemic treatments as well as more frequent screening and imaging with MRI. BMs may cause multiple symptoms, significantly impacting the quality of life (QoL), function, and symptoms [1]. As BMs are associated with limited life expectancy, a systematic evaluation and follow-up of symptoms is important to preserve QoL, tailor follow-up care, and avoid futile treatment [2]. This is also recommended in treatment guidelines in oncology and palliative care [3,4]. Furthermore, it is a key component of patient-centered care, where the patients’ own perception of symptoms and functioning should be considered in the decision-making processes. Notably, integrating palliative care with systematic symptom follow-up both improved symptom control and survival in NSCLC patients, regardless of BM status [5].

Although selected groups of NSCLC patients with BMs may be candidates for surgical resection or targeted therapy, radiotherapy (RT) is the most frequently offered treatment option. The purpose of RT is to control intracranial disease to reduce symptom intensity, maintain or improve quality of life and function, and potentially prolong survival. However, RT frequently causes acute and long-term side effects that may impact QoL [6,7]. Stereotactic radiotherapy (SRT) has become the preferred RT modality for patients with one to four BMs due to better preservation of cognitive function compared to whole brain radiotherapy (WBRT). WBRT is mostly offered to patients with five or more BMs who are not candidates for SRT or systemic treatment [8,9]. However, WBRT should be questioned in patients with short expected survival, as the QUARTZ study found similar survival and quality-adjusted life-years when comparing WBRT to the best supportive care in patients unsuitable for surgical resection or SRT [10].

Patient-reported outcomes (PROs) of level of function and symptom severity provide information about the patients’ perceived health status, symptom burden, and level of function. This information is important for treatment decisions and follow-up [11]. A prospective study validated the combination of two questionnaires (EORTC QLQ-C15-PAL and QLQ-BN20) in patients with BMs (58% lung cancer, 55% treated with WBRT only) [12]. After one month, overall QoL and most symptoms remained stable, with a few exceptions of improvements (future uncertainty, insomnia, visual disorders, communication deficit, and drowsiness). In contrast, a systematic review of NCSLC patients with BMs found that WBRT generally resulted in stable or worsened QoL scores rather than improvements [7]. A systematic review of the literature prior to 2022 found no significant change in QoL after SRT, but patients with poor physical function and advanced cancer disease before SRT reported worsened QoL after SRT [13].

There are few recent prospective real-life studies reporting PROs in NSCLC patients with BMs treated with RT, particularly in patients with an expected short survival. Knowledge of real-life data regarding overall QoL in patients with brain metastases was called for by the National Cancer Institute Collaborative Workshop on Shaping the Landscape of Brain Metastases in 2023 [14]. Here, we present PROs from the start of RT and at month 2 after the start of RT in the NSCLC cohort of our large BM study, “Brain metastases in Norway—A Prospective Cohort Study” [15]. By providing more information on overall QoL, function, and symptoms before and after RT, we aim to identify patient groups with little evidence for improvement after RT in overall quality of life, physical function, fatigue, and dyspnea. From a clinical point of view, we consider these as essential QoL items for lung cancer patients. In this way, our (ultimate) goal is to facilitate treatment decision making and reduce futile RT. The following research questions apply: Can PROs at the start of RT be useful in survival estimates, and how do PROs change over time after RT?

## 2. Materials and Methods

### 2.1. Identification

Study design and identification of eligible patients in the prospective cohort study “Brain metastases in Norway—A Prospective Cohort Study” have been described previously (Supplementary Figure S1) [16]. From November 2017 to March 2021, 407 lung cancer patients newly diagnosed with BMs were included in the prospective study; 294 of these had NSCLC, received RT, and were included in the present study.

### 2.2. Patient Characteristics

Details on patient characteristics were extracted as described previously [16]. Status of the primary tumor was defined as controlled (present and stable or removed) or uncontrolled (progressive/synchronous/unknown). Extracranial metastases (ECMs) were defined according to the most recent CT scan at the time of inclusion as controlled (no metastases or stable) or uncontrolled (progressive/synchronous/unknown status). If no brain MRI was available, the number of BMs was based on diagnostic CT. PD-L1 positivity was defined as ≥1% [17]. For PD-L1 testing, we used the PD-L1 clone 22C3 pharmDx kit from Dako (Glostrup, Denmark).

### 2.3. Treatment

The RT was performed on a linear accelerator system (LINAC). WBRT was given as two opposing fields as 20 gray (Gy) in five fractions or 30 Gy in ten fractions. SRT was given in 1–5 fractions with 3–25 Gy per fraction. Neither a gamma-knife nor cyber was used, as these RT modalities were not available in the two healthcare regions.

### 2.4. Patient-Reported Outcome Measures

The EORTC QLQ-C15-PAL is a shortened version of the validated EORTC QLQ-C30 developed to reduce the assessment burden on patients with limited life expectancy [18]. The questionnaire consists of 15 questions: two multi-item function scales (physical and emotional function), two multi-item symptoms scales (fatigue and pain), five single-item symptom scales (dyspnea, sleep disturbance, appetite loss, nausea/vomiting, and constipation), and one regarding overall QoL.

The EORTC QLQ-BN20 was initially developed for patients with primary brain tumors but has been validated in patients with BMs [12]. The questionnaire consists of 20 questions that constitute four multi-item symptom scales and seven single-item symptoms. In this study, three multi-item scales (visual disorder, communication deficit, and motor dysfunction) and four single-item symptom scales (headache, seizures, drowsiness, weakness of legs) were used.

Both questionnaires were completed at the start of RT (M0), and subsequent forms were sent by mail every month up to one year or death. When a questionnaire was not returned, one additional form was sent by mail as a reminder to keep missing data at a minimum. The patient rated 14 of the questions from 1 (not at all) to 4 (very much) and overall QoL as 1 (poor) to 7 (excellent). Item scores were linearly transformed to 0–100 scales according to the conventional EORTC algorithm, with higher scores representing more symptoms or better function and overall QoL [19].

### 2.5. Statistical Analysis

Overall survival was estimated by Kaplan–Meier analyses from the start of RT for BMs to death or last follow-up, 1 October 2023. Descriptive statistics were used for patient characteristics and PROs. Patients who completed questionnaires at the start of RT (M0) were classified as “responders”, otherwise as “non-responders”. In the analysis of PRO scores, responders who completed questionnaires both at the start of RT and at month 2 (M2) were analyzed in groups according to survival after the start of RT (<6 months: short-term survivors; >6 months: long-term survivors) [16]. In a second analysis, patients were grouped according to ECOG status at inclusion (ECOG 0–1, ECOG 2, and ECOG 3–4).

All PRO items were analyzed, but with a special focus on overall QoL, physical function, fatigue, and dyspnea, reflecting our clinical experience with NSCLC patients. Mean PRO scores are reported with standard deviations (SDs). The difference in mean PRO scores at M0 and at M2 after the start of RT is presented with a 95% confidence interval (95% CI). Month 2 was selected for comparison due to a high attrition rate at later time-points. A difference in mean scores ≥10 points in the transformed 0–100 scales was defined as a clinically meaningful difference [20]. Missing data in the EORTC questionnaires were processed in accordance with the EORTC scoring algorithms [19].

Uni- and multivariable survival analyses were performed using the Cox proportional hazard model. A threshold for “poor function” was set at scores ≤66.7 for function scales and scores ≥33.3 for “high symptom burden” for symptom scales. PROs with a *p* < 0.001 in univariable analyses were tested one-by-one/stepwise in the adjusted Cox models. All analysis were performed by SPSS Statistics 29 (IBM Corp., Armonk, NY, USA).

### 2.6. Ethical Considerations

This study was approved by the Regional Committees of Medical and Health Research Ethics in each participating health region (reference no. 2017/1358). Authorization for data access was approved by the Data Protection Agency (reference No.18/05943). All data were stored and handled according to General Data Protection Regulation (GDPR).

## 3. Results

### 3.1. Patient Characteristics

Of the 294 NSCLC patients treated with RT, 239 (81%) completed questionnaires at the start of RT (responders) (Figure S1). Of these, 40% (n = 96) completed questionnaires only at the start of RT (M0). Further, 44% (n = 105) responded both at M0 and M2, as shown in Table 1. Compared to responders (median age 69 years (35–84)), non-responders (n = 55, median age 72 years (53–92)) had a lower proportion of patients with ≥5 BMs (22% vs. 44%) and patients treated with WBRT (31% vs. 52%), as shown in Table 1.

Responders at M0 only had poorer ECOG status (ECOG 0–1 38% vs. 69%), had less often controlled ECM (31% vs. 50%), and were more likely to be treated with WBRT (59% vs. 42%) compared to patients responding both at M0 and M2 (Table 1).

Of the 239 responders, 138 (58%) survived <6 months (short-term survivors). Compared to the 101 (42%) long-term survivors (survival >6 months), a lower percentage of short-term survivors had ECOG 0–1 (38% vs. 77%) and controlled ECM (30% vs. 55%), whereas a higher percentage had ≥5 BMs (44% vs. 32%) and received WBRT (62% vs. 39%) (Table 1).

### 3.2. Survival

Thirty-three patients were alive at last follow up (1 October 2023), with a median follow-up time of 51.0 months (range 27.0–64.6). Overall, 41% of the patients died within 3 months and 58% within 6 months, while 30% were alive 1 year after the start of RT. For responders (n = 239), median overall survival (mOS) after the start of RT was 4.4 months (95% CI 3.4–5.4) compared to 4.0 months (2.5–5.5) for non-responders. For patients completing questionnaires at the start of RT only, mOS was 1.4 months (95% CI 1.2–1.7) compared to 9.4 months (6.1–12.7) in patients who completed questionnaires at later time-points. For short-term survivors, mOS was 1.8 months (95% CI 1.5–2.2) compared to 24.9 months (95% CI 18.1–31.8) for long-term survivors.

### 3.3. PROs

In the 239 patients responding at the start of RT (M0), the response rate declined gradually: 62% (132/214 patients alive) at month 1, 63% (105/166) at month 2, 60% (61/101) at month 6, and 52% (38/73) at month 12 (Table S1).

#### 3.3.1. Association Between PROs at Start of Radiotherapy and Survival

In the univariable survival analyses, ECOG 2–4, uncontrolled ECM, PF, motor dysfunction, and weakness of legs were significantly associated with survival time (*p* < 0.001), as shown in Table 2. In a stepwise Cox multiple regression model, adjusting for ECOG 2–4 and uncontrolled ECM, only weakness of legs remained associated with survival (HR 1.5 [95% CI 1.1–2.0], *p* = 0.008).

#### 3.3.2. Patients Completing Questionnaires at M0 Only

Patients completing questionnaires at M0 only had worse mean scores (difference in mean score ≥ 10) for overall QoL and physical function, a similar mean score for fatigue, and a borderline worse mean score for dyspnea (difference 9.7) compared to those with subsequent completion of PROs (Table 3). They also reported worse mean scores for pain, loss of appetite, constipation, motor dysfunction, and weakness of legs. For the remaining domains, mean scores were similar (difference in mean score < 10).

#### 3.3.3. Patients Completing Questionnaires at Both M0 and M2

At M2, the total group of responders (n = 105) reported worsened PF, nausea/vomiting, loss of appetite, and weakness of legs compared to M0 (Table 3). For the remaining domains, mean scores were similar.

**Figure 1:**
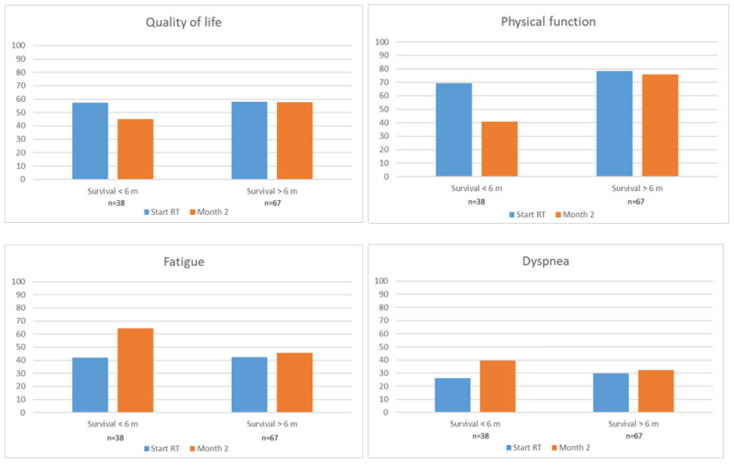
Patient reported outcome scores (mean) at start of RT (M0) and month 2 (M2) by survival time. Only patients responding at both M0 and M2 were included in the analyses. Higher scores for quality of life and physical function indicate better function. Higher scores for fatigue and dyspnea indicate worse symptoms.

Of those responding at both M0 and M2, 38 (36%) were short-term survivors and 67 (64%) were long-term survivors. There were no differences in mean scores for overall QoL, PF, fatigue, and dyspnea between short-term and long-term survivors at M0 (Table S2 and Figure 1). For the remaining domains, mean scores were also similar except for more constipation and borderline more weakness of legs in the short-term survivors (28.9 vs. 19.2). At M2, short-term survivors had worsened mean scores for overall QoL, physical function, fatigue, and dyspnea compared to at M0. They also reported worse mean scores for nausea/vomiting, pain, loss of appetite, motor dysfunction, drowsiness, and weakness of legs. The remaining domains were stable, and no mean scores improved. In contrast, at M2, long-term survivors reported stable mean scores for all domains, except for better sleep, compared to at M0 (Table S2 and Figure 1).

#### 3.3.4. PROs According to ECOG Status

**Figure 2:**
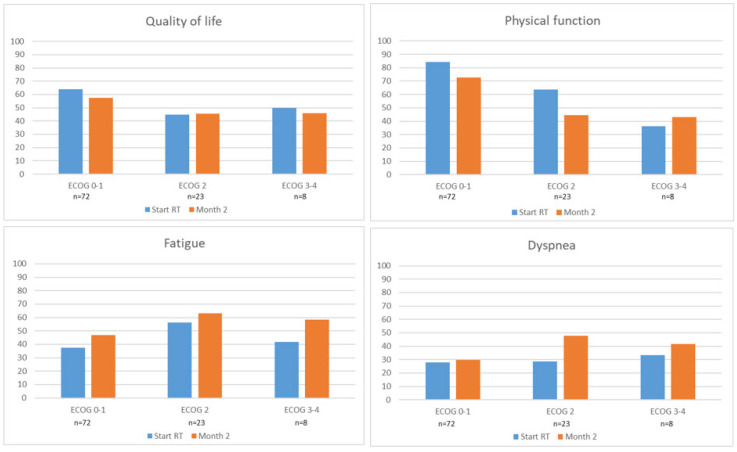
Patient-reported outcome scores (mean) at start of RT (M0) and month 2 (M2) according to ECOG status. Only patients responding at both M0 and M2 were included in the analyses. Higher scores for quality of life and physical function indicate better function. Higher scores for fatigue and dyspnea indicate worse symptoms.

At the start or RT, of those responding at both M0 and M2, patients with ECOG 2 (n = 23, 22%) reported worse mean scores for overall QoL, PF, and fatigue but similar a mean score for dyspnea compared to patients with ECOG 0–1 (n = 72, 70%), as shown in Table S3 and Figure 2. Patients with ECOG 3–4 (n = 8, 8%) reported worse PRO mean scores for overall QoL and PF but similar mean scores for fatigue and dyspnea compared to the ECOG 0–1 patients. When comparing scores between the ECOG 2 and ECOG 3–4 groups, the first had worse mean score for fatigue, better PF, and similar mean scores for QoL and dyspnea.

At M2, mean overall QoL scores remained stable for all ECOG groups. Patients with ECOG 0–1 at M0 reported worsened PF and more fatigue but had stable scores for dyspnea (Figure 2). Patients with ECOG 2 reported worsened PF and more dyspnea but stable scores for fatigue, whereas patients with ECOG 3–4 reported worsened fatigue but stable scores for PF and dyspnea. Of note, at M2, patients with ECOG 2 before the start of RT reported deterioration in more domains than patients with ECOG 0–1.

#### 3.3.5. PROs According to Number of BMs

At the start of RT, there were no differences in mean PRO scores between patients with 1 BM, 2–4 BMs, and ≥5 BMs (Table S4). At month 2, patients with 1 BM reported stable mean PRO scores. Patients with 2–4 and ≥5 BMs reported worsened PF and more fatigue compared to at M0. Also, patients with ≥5 BMs reported borderline worse mean score for dyspnea. At M2, patients with ≥5 BMs reported worse scores for PF and fatigue compared to patients with 1 BM.

## 4. Discussion

### 4.1. Major Findings

This study presents patient reported outcomes (PROs) in a real-life cohort of 239 NSCLC patients treated with radiotherapy (RT) for brain metastases (BM). Except for weakness of legs, PRO scores at the start of RT did not add prognostic information. Two months after RT, short-term survivors reported worsened function and higher symptom burden than at the start of RT, while long-term survivors did not report clinically significant changes.

### 4.2. Multivariable Analyses

It would be useful for clinical decision making to identify PROs that could add prognostic information, as demonstrated in a study of patients with advanced melanoma [21]. Such information could help better identify BM patients with an expected short-term survival and as such be an important factor to consider in treatment decision making. Previous research from our group has shown that ECOG 2–4 and uncontrolled ECMs are associated with poor survival in this NSCLC cohort [16]. When adjusting for these factors in a step-wise, multivariate analysis, PRO scores at the start of RT were not associated with survival except for high symptom scores for weakness of legs. Based on the present results, a high symptom score on weakness of legs (i.e., high symptom burden) may add prognostic information, perhaps capturing more information on functioning than what is captured in ECOG status estimations alone.

### 4.3. PRO Scores

Grouping patients according to their response patterns revealed differences in clinical characteristics and survival time after RT between response groups. Patients who responded at the start of RT (M0) only reported worse mean PRO scores at M0 and had shorter survival compared to patients who also responded at later time points. Shorter survival in the group responding at M0 only was consistent with a higher frequency of clinical factors associated with poor survival (i.e., ECOG 2–4 and uncontrolled extracranial metastases) in this group. When comparing short-term and long-term survivors who responded at both M0 and M2, there was a higher frequency of poor prognostic factors among those with a short survival time.

At M0, short-term and long-term survivors reported similar mean PRO scores. At M2, short-term survivors reported worsened function and higher symptom burden compared to at M0, whereas long-term survivors reported stable mean PRO scores. These findings are consistent with a prospective study by Otto-Vollaar et al. [22]. Here, patients (54% lung cancer) were asked whether they experienced benefit from RT two months after WBRT. Median OS was 2.9 months for patients who reported no benefit from WBRT; these patients also reported decreased QoL at two months. In contrast, mOS was 8.1 months in patients who reported a benefit of RT; this group reported increased QoL. Similar to our study, they found no difference in mean PRO scores between the two groups at the start of RT. Steinmann et al. found worsened PRO scores three months after WBRT in patients with a median OS of 4.5 months, comparable to our short-term survivors (mOS 1.8 months) [6]. A recent prospective study (61% lung cancer) found no change or improvement in QoL 6 months after SRT in patients with a mOS of 27.3 months, which is comparable to our long-term survivors (mOS of 24.9 months) [23]. Stable PRO scores in long-term survivors may be partly affected by response shift, which occurs when patients adjust to cope with their disease and symptoms [24].

The majority of cancer patients die from progressive extracranial disease [25]. As a result, one may assume that patients experience worsened function and higher symptom burden as they approach death. Additionally, BM patients frequently experience acute negative side effects from intracranial RT. At M0, the majority of short-term survivors had ECOG 2–4 (62%) and uncontrolled ECMs (70%), factors that are associated with a poor prognosis. On the other hand, among the long-term survivors, 21% had ECOG 2–4 and 45% had uncontrolled ECMs. The worse mean PRO scores at M2 for short-term survivors compared to long-term survivors are most likely due to differences in clinical status before RT that may also influence their tolerance for RT’s side effects. This supports our suggestion to avoid RT in NSCLC patients with an expected short survival (i.e., those with ECOG 2–4 and uncontrolled ECMs) [16]. These patients should be offered palliative care with a systematic symptom follow up rather than RT. This is consistent with recommendations in the QUARTZ study, which found no clinical or survival benefit from adding WBRT to the best supportive care in patients with NSCLC who were not candidates for surgery or SRT [10]. In contrast, long-term survivors (patients with good prognostic characteristics at M0) appear to benefit from RT, as their PRO scores remain stable after RT. Additionally, long-term survivors are more likely to be candidates for systemic treatment after RT, potentially contributing to even longer survival [26].

Patients who responded at M0 only had worse mean PRO scores than patients who responded also at M2. This finding is consistent with those of Steinmann et al., who found worse mean PRO scores in patients who responded before the start of RT (56%) only [27]. As in our study, these patients had a poorer performance status than those who responded at later time points. In contrast, Komosinska et al. found no difference in baseline mean scores between patients who only completed the first questionnaire and those who completed the questionnaire one month after WBRT (65% lung cancer) [28]. All patients in Komosinska et al.’s study had KPS ≤ 60 (ECOG 2–4), which could explain the lack of difference between the groups. Patients who responded at M0 only and those who were short-term survivors had similar clinical characteristics. It is the fittest patients that are likely to respond over time [29]. Short-term survivors had worsened mean PRO scores at M2, and it is likely that patients who did not respond after M0 experienced even worse function and a higher symptom burden at that time point than those that did respond. This strengthens our recommendation to refrain from RT in NSCLC patients with BMs and a short expected survival [16].

Different patterns in mean PRO scores at M0 and two months after RT were found when grouping patients according to ECOG status at the start of RT. At M0, patients with ECOG 0–1 had better mean PRO scores than those with ECOG 2–4. This is consistent with Caissie et al.’s finding that patients with a higher performance status had better PF and overall QoL before RT [12]. Furthermore, from the start of RT to M2, overall QoL remained stable across ECOG groups, despite all groups having worsened PRO scores in one or more domains. As mentioned above, stable overall QoL scores might be explained by a response shift [24]. That patients with ECOG 2 before the start of RT reported worse scores in more domains at M2 than those with ECOG 0–1 supports that RT should be questioned in patients with ECOG 2–4, despite challenges with concluding specifically for the small ECOG 3–4 group.

Different patterns in mean PRO scores at M0 and at month 2 were also found when grouping patients according to number of BMs at the start of RT. At M0, mean PRO scores were similar among patients with 1 BM, 2–4 BMs, and ≥5 BMs. Worsened PRO scores at month 2 in patients with multiple BMs may reflect a higher intracranial tumor burden and that these patients are more likely to be treated with WBRT. This finding also corresponds to the fact that almost two-thirds of these patients were short-term survivors who reported worsened function and higher symptom burden at month 2.

### 4.4. Strengths and Limitations

Some strengths and limitations of this population-based prospective cohort study have already been discussed [16]. A relatively large sample size and a real-life cohort is a clear strength of this study. Other strengths include assessment of questionnaires at early time points and monthly after RT. To our knowledge, this is one of few prospective studies which report PROs of patients that complete questionnaires at the start of RT only. Patients who do not complete follow-up questionnaires are usually excluded from study analyses due to difficulties with interpretation of data and statistical analyses. Moreover, patients with characteristics associated with short survival (ECOG 2–4 and uncontrolled ECM) are often ineligible for clinical studies [30]. As a result, important information about many real-life patients is often missing.

It is also a strength that responders and non-responders were similar in terms of clinical characteristics, except there were more patients with 1–4 BMs and more use of SRT in the non-responders. This indicates that the study findings may only to a small extent be affected by selection bias, resulting in a good internal validity. Nevertheless, a high attrition rate is the major limitation of this study, although it corresponds to other studies in patients with advanced cancer [6,12,22]. A high attrition rate can first and foremost be explained by the fact that 40% of the patients died within three months, reflecting the vulnerability of NSCLC patients with BMs. Using the short EORTC QLQ-C15-PAL and selected items of the EORTC QLQ-BN20 to shorten the questionnaire completion time and lessen the burden may have counteracted even higher attrition.

## 5. Conclusions

Patients who died within 6 months after RT for BMs reported worsened PRO scores two months after RT. This indicates that patients diagnosed with BMs and a short expected survival—those with ECOG 2–4 and uncontrolled extracranial disease—do not benefit from RT and should rather be offered optimal palliative care. Patients with a longer expected survival (>6 months) appear to benefit from RT. Upon determining expected survival, clinicians can use our findings on PRO data after RT to supplement information to patients about their likely trajectory and expected quality of life. Further studies investigating PROs as prognostic factors at the time of brain metastases diagnosis are warranted.

## Figures and Tables

**Table 1:** Patient characteristics at start of RT.

Characteristic	Responders n = 239 (%)	Non-Responders n = 55 (%)	Responders at M0 Only n = 96 (%)	Responders at Both M0 and M2 n = 105 (%)	Short-Term Survivors (<6 Months) n = 138 (%)	Long-Term Survivors (>6 Months) n = 101 (%)
**Gender**						
Male	122 (51)	23 (42)	49 (51)	51 (49)	72 (52)	50 (50)
**Age**						
<70	127 (53)	25 (46)	48 (50)	58 (55)	68 (49)	59 (58)
≥70	112 (47)	30 (54)	48 (50)	47 (45)	70 (51)	42 (42)
**ECOG**						
ECOG 0–1	131 (55)	27 (49)	36 (38)	72 (69)	53 (38)	78 (77)
ECOG 2	70 (29)	15 (27)	36 (38)	23 (22)	54 (39)	16 (16)
ECOG 3–4	35 (15)	12 (22)	23 (24)	8 (7)	30 (22)	5 (5)
Unknown	3 (1)	1 (2)	1	2(2)	1 (1)	2 (2)
**Histology**						
Adenocarcinoma	180 (75)	40 (73)	69 (72)	85 (81)	94 (68)	86 (85)
Squamous cell carcinoma	34 (14)	10 (18)	15 (16)	12 (11)	22 (16)	12 (12)
Others *	25 (11)	5 (9)	12 (12)	8 (8)	22 (16)	3 (3)
Mutation status/PD-L1 status **						
Present (EGFR = 16, ALK = 4, PD-L1 = 129)	136 (57)	30 (55)	50 (52)	59 (56)	75 (54)	61 (60)
Absent	103 (43)	25 (45)	46 (48)	46 (44)	63 (46)	40 (40)
**Clinical status primary tumor**						
Primary tumor controlled/removed	69 (29)	17 (31)	25 (26)	35 (33)	36 (26)	33 (33)
Uncontrolled	170 (71)	38 (69)	71 (74)	70 (67)	102 (74)	68 (67)
**Extracranial metastases**						
Controlled	97 (41)	27 (49)	30 (31)	52 (50)	41 (30)	56 (55)
Uncontrolled	142 (59)	28 (51)	66 (69)	53 (50)	97 (70)	45 (45)
**Number of BMs**						
1	70 (29)	19 (35)	29 (30)	32 (31)	33 (24)	37 (36)
2–4	76 (32)	24 (43)	25 (26)	38 (36)	44 (32)	32 (32)
≥ 5	93 (39)	12 (22)	42 (44)	35 (33)	61 (44)	32 (32)
Largest diameter of BMs						
<3 cm	163 (68)	39 (71)	62 (65)	74 (70)	89 (65)	74 (73)
≥3 cm	68 (29)	12 (22)	31 (32)	27 (26)	44 (32)	24 (24)
Missing	8 (3)	4 (7)	3 (3)	4 (4)	5 (3)	3 (3)
**Initial treatment**						
WBRT	124 (52)	17 (31)	57 (59)	44 (42)	85 (62)	39 (39)
SRT	115 (48)	38 (69)	39 (41)	61 (58)	53 (38)	62 (61)

* Large cell ccarcinoma, unknown, others. ** PD-L1 positivity defined as >1%.

**Table 2:** Prognostic factors for overall survival.

	Univariable			Multivariable					
	n	HR (95% CI)	*p* Value	HR (95% CI)	*p* Value	HR (95% CI)	*p* Value	HR (95% CI)	*p* Value
**Baseline characteristic and PROs**									
**ECOG**									
ECOG 0–1	131	1		1		1		1	
ECOG 2–4	105	3.0 (2.2–4.0)	<0.001	2.5 (1.8–3.6)	<0.001	2.8 (2.0–3.7)	<0.001	2.7 (2.0–3.7)	<0.001
Missing	3								
**ECM**									
Controlled	96	1		1		1		1	
Uncontrolled	140	1.9 (1.4–2.5)	<0.001	1.9 (1.4–2.6)	<0.001	2.0 (1.5–2.6)	<0.001	1.9 (1.4–2.5)	<0.001
**BMs**									
1–4 BMs	145	1							
≥ 5	91	1.5 (1.1–2.0)	0.005						
Largest diameter of BMs									
<3 cm	163	1							
≥3 cm	68	1.4 (1.0–1.8)	0.044						
Missing	8								
**Clinical status primary tumor**									
Controlled/removed	69	1							
Uncontrolled	167	1.1 (0.8–1.5)	0.622						
EORTC QLQ-C15 PAL									
**Function scales**	(>66.7/≤66.7) ^a^								
Overall QoL	54/177 ^c^	1.2 (0.9–1.7)	0.279						
Physical function	115/117 ^d^	2.1 (1.6–2.8)	<0.001	1.3 (1.0–1.9)	0.073				
Emotional function	126/107 ^e^	1.2 (0.9–1.5)	0.271						
**Symptom scales**	(<33.3/≥33.3) ^b^								
Fatigue	50/183 ^f^	1.3 (0.9–1.9)	0.112						
Nausea/vomiting	155/77 ^g^	1.2 (0.9–1.6)	0.294						
Pain	121/111 ^h^	1.6 (1.2–2.1)	0.002						
Dyspnea	80/153 ^i^	1.1 (0.8–1.5)	0.486						
Sleep disturbance	89/144 ^j^	0.8 (0.6–1.0)	0.101						
Appetite loss	133/99 ^k^	1.4 (1.1–1.9)	0.014						
Constipation	104/127 ^l^	1.4 (1.0–1.8)	0.040						
EORTC QLQ-BN20									
Headaches	136/98 ^m^	0.9 (0.7–1.2)	0.333						
Visual disorder	179/55 ^n^	1.4 (1.0–1.9)	0.035						
Seizures	201/33 ^o^	1.1 (0.8–1.6)	0.632						
Motor dysfunction	133/103	1.7 (1.3–2.2)	<0.001			1.3 (1.0–1.8)	0.053		
Communication deficit	189/46 ^p^	1.3 (1.0–1.9)	0.093						
Drowsiness	56/177 ^q^	1.5 (1.1–2.2)	0.011						
Weakness of legs	101/132 ^r^	1.8 (1.4–2.4)	<0.001					1.5 (1.1–2.0)	0.008

^a^ Score >66.7/≤66.7; ^b^ Score <33.3/≥33; ^c^ Six patients not available, ^d,g,k^ Four patients not available, ^e,f,h,i,j,q,r^ Three patients not available, ^l^ Five patients not available, ^m,n,o^ Two patients not available, ^p^ One patient not available.

**Table 3:** Mean scores (SD) of EORTC QLQ-C15 PAL and EORTC QLQ-BN20 for all responders, patients responding only at start of RT (M0), and patients responding at both M0 and M2.

EORTC Scale	Responders n = 239	Responders at M0 Only n = 96	Responders Both at M0 and M2 n = 105	
	M0		M0	M2
**EORTC QLQ-C15 PAL**	Mean score (SD)			
Overall QoL	54.1 (25.5)	47.7 (25.3)	58.5 (23.3)	53.6 (22.7)
Physical function	67.0 (27.0)	57.2 (26.8)	75.4 (24.3)	63.9 (30.7)
Emotional function	73.1 (27.1)	70.3 (70.3)	78.8 (23.8)	78.6 (26.5)
Fatigue	44.6 (26.1)	49.5 (27.7)	42.0 (24.5)	51.6 (28.5)
Nausea/vomiting	15.2 (24.9)	20.0 (29.3)	11.8 (20.8)	22.1 (28.5)
Pain	30.5 (31.0)	38.9 (33.7)	24.5 (27.3)	26.9 (30.4)
Dyspnea	33.6 (30.7)	38.5 (33.3)	28.8 (27.4)	34.6 (30.1)
Sleep disturbance	33.9 (33.0)	34.4 (35.4)	34.3 (31.6)	25.6 (29.1)
Appetite loss	23.1 (31.6)	29.9 (34.4)	19.1 (29.6)	34.3 (36.1)
Constipation	27.8 (30.9)	34.7 (33.1)	24.0 (30.4)	26.9 (32.5)
**EORTC QLQ-BN20**				
Headaches	19.5 (26.9)	22.5 (28.9)	18.3 (25.4)	15.7 (22.3)
Visual disorder	15.2 (22.5)	19.8 (27.4)	13.6 (20.3)	15.5 (20.1)
Seizures	6.5 (17.5)	5.9 (17.4)	7.4 (18.0)	7.4 (19.1)
Motor dysfunction	29.1 (25.4)	37.8 (25.4)	22.2 (24.4)	25.1 (26.4)
Communication deficit	15.2 (21.7)	15.9 (20.4)	14.4 (23.6)	13.4 (22.0)
Drowsiness	38.3 (26.7)	42.8 (29.8)	36.2 (28.0)	45.1 (28.5)
Weakness of legs	29.0 (31.6)	38.2 (32.2)	22.8 (28.7)	36.5 (33.5)

## Data Availability

The data from this study may be made available upon request to the corresponding author who will review and seek approval from the study investigators.

## Supplementary Materials

The following supporting information can be downloaded at: https://www.mdpi.com/article/10.3390/cancers17091529/s1, Figure S1: Treatment breakdown; Table S1: Mean scores (SD) of EORTC QLQ-C15 PAL and EORTC QLQ-BN20 for the total number of responders at each time point; Table S2: Patient-reported outcome scores (mean) at start of RT (M0) and month 2 (M2) by survival time; Table S3: Change in PROs from start of RT (M0) to month 2 (M2) for complete responders at M0 and M2 by ECOG status. Table S4: Patient reported outcome scores (mean) at start of RT (M0) and month 2 (M2) by number of BM.
