# Supplementary material for: Patient-Reported Outcomes Before and After Radiotherapy for Brain Metastases—A Prospective Cohort Study of 239 Non-Small-Cell Lung Cancer Patients
Source: Cancers (Basel). 2025 Apr 30;17(9):1529. doi: 10.3390/cancers17091529 (PMC12072175; doi:10.3390/cancers17091529)
Supplement: Supplementary file 1 [file cancers-17-01529-s001.zip › cancers-3602574-supplementary.pdf]

Figure S1. Treatment breakdown

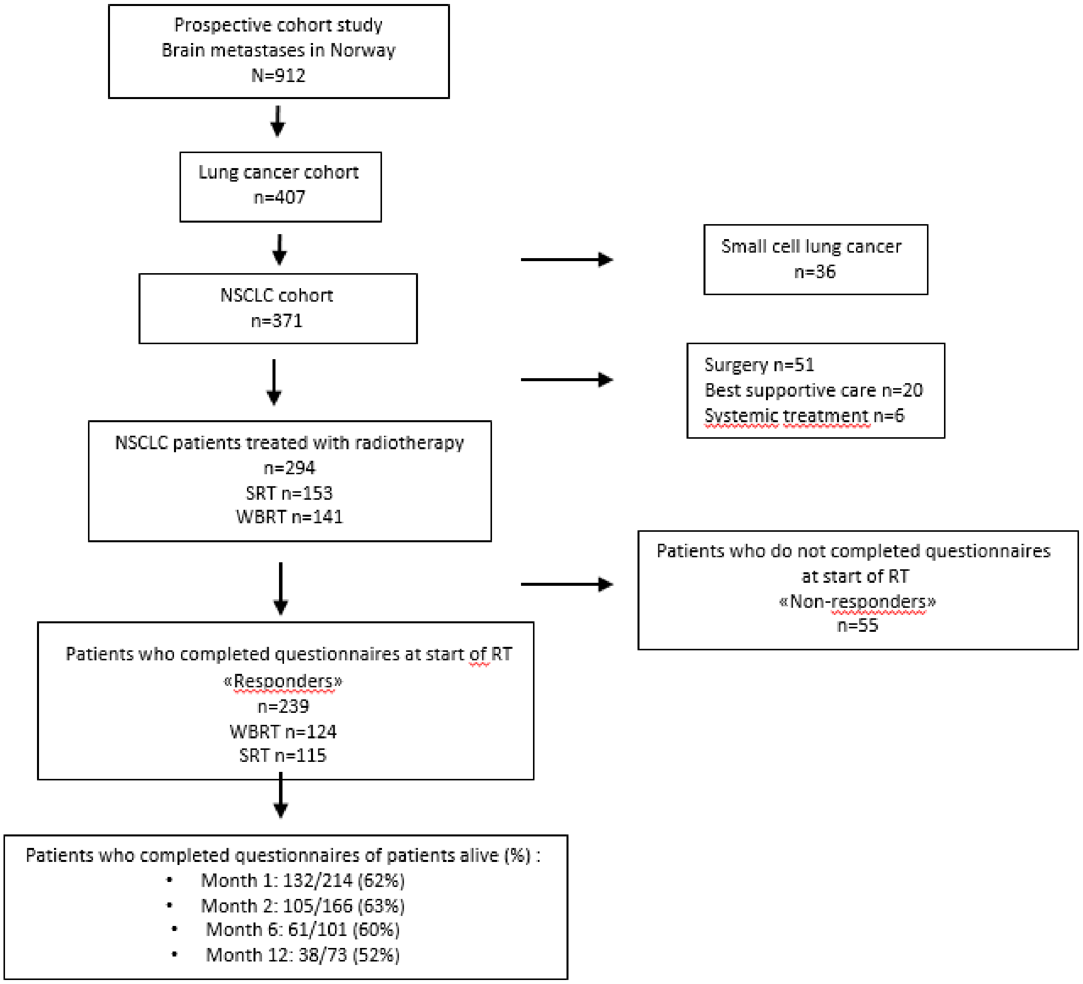

Table S1. Mean scores (SD) of EORTC QLQ-C15 PAL and EORTC QLQ-BN20 for the total number of responders at each time point.

	Start of RT	***Month 1***	***Month 2***	***Month 6***	***Month 12***
**Responders/alive (%)**	239/239 (100%)	132/214 (62%)	105/166 (63%)	61/101 (60%)	38/73 (52%)
EORTC scale					
EORTC QLQ-C15 PAL	Mean score (SD)				
Overall QoL	54.1 (25.5)	56.2 (24.1)	53.6 (22.7)	61.5 (24.8)	66. 2(20.2)
Physical function	67.0 (27.0)	67.1 (26.7)	63.9 (30.7)	79.8 (22.5)	78.7 (23.3)
Emotional function	73.1 (27.1)	76.4 (27.6)	78.6 (26.5)	80.1 (23.7)	82.9 (18.8)
Fatigue	44.6 (26.1)	52.8 (27.5)	51.6 (28.5)	35.0 (25.6)	31.6 (23.5)
Nausea/vomiting	15.2 (24.9)	17.3 (24.7)	22.1 (28.5)	14.2 (24.7)	12.3 (16.3)
Pain	30.5 (31.0)	25.5 (29.1)	26.9 (30.4)	19-4 (26.6)	15.8 (22.6)
Dyspnea	33.6 (30.7)	36.7 (32.4)	34.6 (30.1)	26.7 (26.6)	21.1 (25.0)
Sleep disturbance	33.9 (33.0)	30.0 (29.49	25.6 (29.1)	21.3 (25.8)	19.3 (25.3)
Appetite loss	23.1 (31.6)	29.0 (33.3)	34.2 (36.1)	19.7 (29.4)	23.7 (29.9)
Constipation	27.8 (30.9)	26.4 (32.3)	26.9 (32.5)	25.7 (33.6)	27.1 (25.5)
EORTC QLQ-BN20					
Headaches	19.5 (26.9)	16.9 (23.5)	15.7 (22.3)	17.5 (23.3)	11.4 (19.4)
Visual disorder	15.2 (22.5)	14.4 (20.5)	15.5 (22.3)	9.3 (14.0)	8.8 (14.4)
Seizures	6.5 (17.5)	12.6 (25.2)	7.4 (19.1)	7.6 (20.5)	2.6 (12.0)
Motor dysfunction	29.1 (25.4)	25.0 (24.5)	25.0 (26.4)	16.3 (19.4)	15.8 (14.7)
Communication deficit	15.2 (21.7)	13.3 (20.9)	13.5 (22.0)	6.2 (11.6)	7.0 (14.7)
Drowsiness	38.3 (26.7)	44.2 (27.9)	45.1 (28.5)	33.9 (26.4)	29.8 (21.6)
Weakness of legs	29.0 (31.6)	39.9 (33.1)	36.5 (33.5)	23.5 (23.8)	21.9 (24.8)

Table S2. Patient reported outcome scores (mean) at start of RT (M0) and month 2 (M2) by survival time. Only patients responding at both M0 and M2 were included in the analyses. Short-term survivors (survival < 6 months) n=38. Long-term survivors (survival > 6 months) n=67.

EORTC Scale	*Groups*	*Start of RT*	*Month 2*	*Mean change (CI) Start of RT vs. Months 2*	*Change ≥ 10 points*
EORTC QLQ-C15 PAL		Mean score (SD)			
Overall QoL	Total group	57.7 (22.8)	53.6 (22.7)	-4.7 (-9.9,0.5)	
	Short-term survivors	57.4 (22.3)	44.9 (23.5)	-12.5 (-22.7,-2.3)	x
	Long-term survivors	57.9 (23.79	57.7 (21.1)	-0.2 (-5.9,5.4)	
Physical function	Total group	75.2 (24.3)	63.2 (30.8)	12.0 (-18.0,-6.0)	x
	Short-term survivors	69.4 (23.7)	40.7 (31.9)	-28.7 (-40.1,-17.3)	x
	Long-term survivors	78.5 (24.79	76.0 (21.5)	-2.5 (-8.3,3.4)	
Emotional function	Total group	78.8 (23.8)	78.6 (26.5)	1.3 (-3.6,6.3)	
	Short-term survivors	80.1 (21.1)	74.8 (29.0)	-5.4 (-14.0, 3.1)	
	Long-term survivors	76.7 (25.4)	80.6 (25.4)	5.3 (-0.7,11.3)	
Fatigue	Total group	42.0 (24.5)	51.6 (28.5)	10.4 (4.0, 16.8)	x
	Short-term survivors	41.9 (24.1)	64.4 (29.2)	22.5 (10.2,34.9)	x
	Long-term survivors	42.2 (25.0)	45.6 (25.4)	3.4 (-3.4,10.2)	
Nausea/vomiting	Total group	11.8 (20.8)	22.1 (28.5)	10.6 (4.8,16.3)	x
	Short-term survivors	9.9 (19.0)	22.5 (32.4)	12.6 (1.1-24.1)	x
	Long-term survivors	13.0 (21.9)	22.4 (26.6)	9.4 (3.0,15.8)	
Pain	Total group	24.5 (27.3)	26.9 (30.4)	2.8 (-3.6,9.1)	
	Short-term survivors	26.6 (30.0)	36.9 (37.9)	10.4 (-2.1,22.9)	x
	Long-term survivors	23.7 (25.9)	22.1 (24.1)	-1.6 (-8.6,5.5)	
Dyspnea	Total group	28.8 (27.4)	34.6 (30.1)	6.6 (0.7,12.5)	
	Short-term survivors	26.1 (27.4)	39.6 (32.2)	13.5 (3.2,23.8)	x
	Long-term survivors	29.7 (27.3)	32.3 (29.1)	2.6 (-4.6,9.8)	
Sleep disturbance	Total group	34.3 (31.6)	25.6 (29.1)	-8.9 (-15.0,-2.8)	
	Short-term survivors	32.4 (32.9)	34.2 (36.4)	1.8 (-6.9,10.5)	
	Long-term survivors	35.4 (31.4)	20.3 (22.7)	-15.1 (-23.1,-7.1)	x
Appetite loss	Total group	19.1 (29.6)	34.3 (36.1)	15.7 (7.0,24.3)	x
	Short-term survivors	18.0 (30.0)	44.1 (40.1)	26.1 (9.1,43.1)	x
	Long-term survivors	20.1 (29.7)	29.6 (33.4)	9.5 (0.1,19.0)	
Constipation	Total group	24.0 (30.4)	26.9 (32.5)	2.7 (-4.1,9.5)	
	Short-term survivors	30.6 (35.5)	37.8 (37.8)	7.2 (-6.4,20.9)	
	Long-term survivors	20.4 (26.6)	20.4 (27.9)	0.0 (-7.5,7.5)	
EORTC QLQ-BN20					
Headaches	Total group	18.3 (25.4)	15.7 (22.3)	-2.6 (-4.1,9.5)	
	Short-term survivors	12.3 (21.1)	12.3 (22.5)	0.0 (-8.4,8.4)	
	Long-term survivors	21.7 (27.1)	17.7 (22.0)	-4.0 (-11.0,2.9)	
Visual disorder	Total group	13.6 (20.3)	15.5 (20.1)	2.0 (-2.0,5.9)	
	Short-term survivors	16.2 (23.8)	21.2 (24.5)	5.0 (-9.3,10.8)	
	Long-term survivors	12.1 (18.2)	12.4 (16.7)	0.3 (-5.1,5.6)	
Seizures	Total group	7.4 (18.0)	7.4 (19.1)	-0.3 (-4.6,3.9)	
	Short-term survivors	10.5 (20.7)	11.4 (24.8)	0.9 (-8.5,10.2)	
	Long-term survivors	5.8 (16.3)	4.7 (14.4)	-1.1 (-5.2,3.1)	
Motor dysfunction	Total group	22.2 (24.4)	25.1 (26.4)	2.8 (-2.2,7.8)	
	Short-term survivors	26.2 (26.1)	37.4 (31.2)	11.3 (1.3,21.2)	x
	Long-term survivors	20.0 (23.3)	18.0 (20.4)	-2.0 (-7.3,3.3)	
Communication deficit	Total group	14.4 (23.6)	13.4 (22.0)	-0.7 (-5.6,4.1)	
	Short-term survivors	15.2 (22.7)	22.1 (27.2)	6.9 (-2.9,16.6)	
	Long-term survivors	13.9 (24.3)	8.8 (16.8)	-5.1 (-10.3,0.1)	
Drowsiness	Total group	36.2 (28.0)	45.1 (28.5)	9.4 (3.0,15.8)	
	Short-term survivors	39.5 (30.9)	57.0 (30.9)	17.5 (4.4,30.7)	x
	Long-term survivors	34.4 (26.3)	39.0 (24.7)	4.6 (-2.1,11.3)	
Weakness of legs	Total group	22.8 (28.7)	36.5 (33.5)	13.5 (6.3,20.6)	x
	Short-term survivors	28.9 (29.2)	54.4 (32.3)	25.4 (12.3,38.5)	x
	Long-term survivors	19.2 (28.1)	25.8 (29.7)	6.6 (-1.6,14.7)	

Table S3. Change in PROs from start of RT (M0) to month 2 (M2) for complete responders at M0 and M2, by ECOG-status.

ECOG 0-1 n=72, ECOG 2 n=23, ECOG 3-4 n=8.

EORTC Scale	ECOG	M0	Month 2	Mean change (CI) M0 vs. M2	≥ 10 points Change
EORTC QLQ-C15 PAL		Mean score			
Overall QoL	ECOG 0-1	63.2	57.1	-6.1 (-12.3,0.0)	
	ECOG 2	43.7	44.4	0.8 (-12.0,13.6)	
	ECOG 3-4	50.0	45.8	-4.2 (-28.6,20.3)	
Physical function	ECOG 0-1	84.3	71.9	-12.4 (-19.4,-5.4)	x
	ECOG 2	62.4	43.4	-19.0 (-34.4,-3.7)	x
	ECOG 3-4	36.1	43.1	6.9 (-17.9,31.7)	
Emotional function	ECOG 0-1	77.7	81.4	3.7 (-2.0,9.3)	
	ECOG 2	80.3	68.2	-12.1 (-23.8,-0.5)	x
	ECOG 3-4	66.7	81.3	14.6 (-5.7,34.9)	x
Fatigue	ECOG 0-1	37.4	48.1	10.6 (3.2,18.1)	x
	ECOG 2	56.1	63.6	7.6 (-8.8,24.0)	
	ECOG 3-4	41.7	58.3	16.7 (-14.9, 48.3)	x
Nausea/ vomiting	ECOG 0-1	10.1	22.7	12.6 (5.8, 19.3)	x
	ECOG 2	15.2	24.2	9.1 (-4.0,22.1)	
	ECOG 3-4	20.8	8.3	-12.5 (-33.2,8.2)	x
Pain	ECOG 0-1	17.9	24.4	6.5 (-0.4,13.5)	
	ECOG 2	36.4	32.6	-3.8 (-21.0,13.4)	
	ECOG 3-4	45.8	35.4	-10.4 (-45.3,24.5)	x
Dyspnea	ECOG 0-1	27.5	30.0	2.4 (-2.8,7.6)	
	ECOG 2	28.8	48.5	19.7 (0.5,38.9)	x
	ECOG 3-4	33.3	41.7	8.3 (-24.1, 40.8)	
Sleep disturbance	ECOG 0-1	29.0	23.7	-5.3 (-12.1,1.5)	
	ECOG 2	50.0	33.3	-16.7 (-33.0, -0.4)	x
	ECOG 3-4	29.1	20.8	-8.3 (-33.0,16.4)	
Appetite loss	ECOG 0-1	16.2	33.3	17.1 (6.9,27.4)	x
	ECOG 2	21.2	43.9	22.7 (1.1, 44.3)	x
	ECOG 3-4	33.3	20.8	-12.5 (-38.0,13.0)	x
Constipation	ECOG 0-1	21.9	24.9	3.0 (-5.2,11.2)	
	ECOG 2	25.8	24.2	-1.5 (-16.3, 13.2)	
	ECOG 3-4	45.8	54.2	8.3 (-30.4,47.0)	
EORTC QLQ-BN20					
Headaches	ECOG 0-1	17.4	16.4	-0.9 (-7.2, 5.3)	
	ECOG 2	18.8	17.4	-1.4 (-14.8,11.9)	
	ECOG 3-4	20.8	8.3	-12.5 (-26.9, 1.9)	x
Visual disorders	ECOG 0-1	11.9	13.2	1.3 (-3.5,6.0)	
	ECOG 2	19.2	24.7	5.6 (-5.1,16.3)	
	ECOG 3-4	12.5	12.5	0.0 (-8.6, 8.6)	
Seizures	ECOG 0-1	3.8	5.7	1.9 (-2.3,6.2)	
	ECOG 2	16.7	13.6	3.0 (-16.6,10.6)	
	ECOG 3-4	16.7	4.1	-12.5 (-33.2,8.2)	x
Motor dysfunction	ECOG 0-1	16.7	19.1	2.3 (-3.1,7.8)	
	ECOG 2	30.7	41.1	10.4 (-1.7,22.5)	x
	ECOG 3-4	50.0	30.6	-19.4 (-45.6,6.7)	x
Communication deficit	ECOG 0-1	10.7	10.8	0.1 (-3.4,3.6)	
	ECOG 2	26.6	24.9	-1.7 (-21.5,18.2)	
	ECOG 3-4	15.3	9.7	-5.6 (-22.8,11.6)	
Drowsiness	ECOG 0-1	30.0	40.0	10.0 (3.1,16.9)	x
	ECOG 2	55.1	55.1	0.0 (-17.9,17.9)	
	ECOG 3-4	37.5	62.5	25.0 (-3.8,53.8)	x
Weakness of legs	ECOG 0-1	15.5	28.6	13.1 (5.1,21.2)	x
	ECOG 2	37.7	52.2	14.5 (-3.3, 32.3)	x
	ECOG 3-4	45.8	50.0	4.2 (-36.5, 44.8)	

Table S4. Patient reported outcome scores (mean) at start of RT (M0) and month 2 (M2) by number of BM. Only patients responding at both M0 and M2 were included in the analyses. 1 BM n=32, 2-4 BM n=38 and ≥5 BM n=35.

	Groups	***Start of RT***	***Month 2***
		Mean score (SD)	
Overall QoL	1 BM	57.8 (21.1)	56.8 (21.1)
	2-4 BM	61.6 (23.8)	54.6 (27.2)
	≥5 BM	55.9 (24.9)	49.5 (18.6)
Physical function	1 BM	71.5 (28.9)	70.8 (26.3)
	2-4 BM	80.2 (29.7)	63.7 (33.4)
	≥5 BM	73.9 (23.8)	57.6 (31.1)
Fatigue	1 BM	45.8 (24.7)	43.2 (27.7)
	2-4 BM	35.2 (22.5)	51.3 (29.6)
	≥5 BM	45.6 (25.4)	59.8 (26.3)
Dyspnea	1 BM	27.1 (24.6)	35.4 (31.6)
	2-4 BM	29.6 (29.6)	30.7 (28.39
	≥5 BM	29.4 (28.1)	38.2 (30.8)
